# Anticoagulant management of cancer-associated thrombosis and thrombocytopenia: a retrospective chart review

**DOI:** 10.1016/j.rpth.2025.102684

**Published:** 2025-01-16

**Authors:** Umaima Abbas, Robin MacKenzie, Ushra Khan, Rija Fatima, Tzu-Fei Wang, Rong Luo, Caroline Hamm, Andrea Cervi

**Affiliations:** 1 Department of Medicine, Schulich School of Medicine & Dentistry, University of Western Ontario, London, Ontario, Canada; 2 Department of Translational Health Science, University of Windsor, Windsor, Ontario, Canada; 3 Department of Medicine, University of Ottawa at The Ottawa Hospital and Ottawa Hospital Research Institute, Ottawa, Ontario, Canada; 4 Department of Mathematics and Statistics, Leddy Library, University of Windsor, Windsor, Ontario, Canada; 5 Department of Biomedical Sciences, University of Windsor, Windsor, Ontario, Canada; 6 Department of Oncology, Windsor Regional Hospital, Windsor, Ontario, Canada

**Keywords:** direct oral anticoagulant, hemorrhage, neoplasms, thrombocytopenia, venous thromboembolism

## Abstract

**Background:**

Patients with cancer-associated thrombosis (CAT) are at an increased risk of recurrent thrombosis and bleeding, especially if there is treatment- or disease-related thrombocytopenia. While direct oral anticoagulants (DOACs) are used in the management of CAT, low molecular weight heparin (LMWH) continues to be recommended for CAT with thrombocytopenia.

**Objectives:**

This study aimed to identify the rates of recurrent venous thromboembolism (VTE) and bleeding in patients with CAT and thrombocytopenia treated with DOACs compared with LMWH.

**Methods:**

A retrospective review of patients with CAT and thrombocytopenia (platelet count <100,000/μL) was conducted. Primary outcomes included rates of recurrent VTE and major bleeding over 90 days.

**Results:**

Forty-two patients met the inclusion criteria; 20 (47.6%) had a solid organ malignancy while 22 (52.4%) had a hematologic malignancy. Within the first 7 days of VTE, 3 (7.1%) patients had a platelet count <25,000/μL, 9 (21.4%) had 25,000 to 50,000/μL, and 19 (45.2%) had 50,000 to 100,000/μL. Sixteen patients (38.1%) received a DOAC for initial treatment, while 19 (45.2%) received LMWH. Among patients treated with DOACs, there were no recurrent VTEs, 2 clinically relevant nonmajor bleeding events (12.5%) within the first 2 weeks, and 1 minor bleed (6.3%) in the second month, while those treated with LMWH had 1 recurrent VTE (5.3%) in the second month and 2 clinically relevant nonmajor bleeding events (10.5%) within the first 2 months.

**Conclusion:**

Rates of thrombosis and major bleeding were similar among thrombocytopenic patients with CAT treated with DOACs and LMWH, although differences in baseline patient characteristics can be confounders. Further prospective research on the optimal anticoagulant management of CAT with thrombocytopenia is needed.

## Essentials

• There is limited data to guide the use of the DOACs in patients with CAT and thrombocytopenia.
• A retrospective review of patients at a large community hospital with CAT and thrombocytopenia was performed to evaluate the safety and efficacy of DOACs relative LMWH.
• Rates of recurrent thrombosis and bleeding were similar among thrombocytopenic patients with CAT treated with DOACs and LMWH.
• Further prospective research on the anticoagulant management of CAT with thrombocytopenia is needed.

## 1 Introduction

Cancer-associated thrombosis (CAT), which is defined as a venous thromboembolic event diagnosed in the setting of active cancer, is the second leading cause of death among patients with cancer [1,2]. Patients with cancer-associated venous thromboembolism (VTE) have worse outcomes than those without cancer, including increased risk of recurrent VTE and anticoagulant-related bleeding owing to multiple factors, including treatment- and disease-related thrombocytopenia [[3], [4], [5]].

Thrombocytopenia complicates the anticoagulant management of cancer-associated thrombosis as it increases the risk of bleeding without reducing the risk of VTE [6,7]. Current guidelines recommend full-dose anticoagulation for patients with CAT and a platelet count >50,000/μL, with the addition of platelet transfusions for patients with high-risk features if platelet count were to be <50,000/μL [8,9]. Although there is established efficacy of direct oral anticoagulants (DOACs) relative to low molecular weight heparin (LMWH) for the treatment of cancer-associated VTE in general, very few patients with platelet counts <100,000/μL were enrolled in the phase 3 randomized controlled trials evaluating DOACs for the management of CAT, and outcomes during periods of thrombocytopenia were not reported [[10], [11], [12]]. Despite limited evidence concerning safety and efficacy, DOACs are variably used to treat patients with CAT and thrombocytopenia in clinical practice. Patient preference for an oral anticoagulant over daily injections and cost are driving factors for the consideration of DOACs in comparison to LMWH [[13], [14], [15]].

Here, we aimed to evaluate the safety and efficacy of DOACs in comparison to LMWH for the management of patients with CAT and thrombocytopenia through a retrospective chart review. Furthermore, we describe patient-, cancer- and treatment-related characteristics in relation to anticoagulant strategy and patient outcomes.

## 2 Methods

### 2.1 Study design and population

A retrospective cohort study of adult patients (aged ≥18 years) with a diagnosis of CAT and thrombocytopenia was conducted at Windsor Regional Hospital, a 500-bed community hospital in southwestern Ontario, between January 1, 2012 and December 31, 2023. CAT was defined as a venous thromboembolic event diagnosed within 6 months of cancer diagnosis or treatment, or metastatic, recurrent, progressive cancer, or hematologic malignancy not in remission. Thrombocytopenia was defined as a platelet count <100,000/μL within 14 days before or after diagnosis of CAT. Data were collected for the first 90 days after VTE diagnosis, with day 1 representing the date of VTE diagnosis. This study was approved by the Windsor Regional Hospital Research Ethics Board (WRH REB #23-459). The review board waived the need for consent.

### 2.2 Data collection and outcomes

University of Windsor REDCap software (version 12.5.4) was used to collect and store patient data from electronic health records. We recorded patient demographics including age at VTE diagnosis, sex, comorbidities, cancer type and treatment, type of VTE, platelet count trends, anticoagulant use, recurrent thrombosis, and bleeding events. Platelet count, anticoagulant management (type, dose, class), platelet transfusion, and platelet transfusion thresholds were recorded at the following time points: at time of VTE diagnosis to day 7, days 8 to 14, days 15 to 30, days 31 to 60, and days 61 to 90. Our primary outcomes included: (1) rates of radiographically confirmed recurrent VTE, and (2) major bleeding events, as defined objectively by the International Society on Thrombosis and Haemostasis criteria [16].

### 2.3 Data analysis

Data were summarized descriptively using mean (SD), median [IQR], and frequency (percentage), where appropriate. The overall small sample size precluded further statistical testing.

## Results

### 3.1 Baseline patient characteristics

Forty-two patients met the inclusion criteria. Four (9.5%) died prior to the study duration of 90 days, while 1 patient (2.4%) was lost to follow-up prior to 90 days due to change in residence.

Baseline patient characteristics are outlined in Table 1. Twenty-two patients (52.4%) were female; median patient age at VTE diagnosis was 65 years (IQR, 56-77). Most patients had a pulmonary embolism at diagnosis (*n* = 17; 40.5%), followed by proximal lower extremity deep vein thrombosis (DVT) (*n* = 16; 38.1%) and catheter-associated upper extremity DVT (*n* = 9; 21.4%). Five (11.9%) patients had a prior history of VTE, and 1 (2.4%) patient had a history of major bleeding.

**Table 1:** Baseline patient characteristics and type of venous thrombosis at diagnosis.

Patient characteristic	Total	DOAC	LMWH	UFH	No anticoagulation
Number of patients, *n* (%)	42 (100)	16 (38.1)	19 (45.2)	6 (14.3)	1 (2.4)
Age, y, median (IQR)	65 (56-77)	66 (57-74)	60 (55-77)	77 (71-78)	61 (0)
Sex, *n* (%)					
Female	22 (52.4)	7 (43.8)	12 (63.2)	2 (33.3)	1 (100)
Male	20 (47.6)	9 (56.3)	7 (36.8)	4 (66.7)	0
Cancer characteristics, *n* (%)					
Solid tumor malignancy	20 (47.6)	7 (43.8)	9 (47.4)	3 (50.0)	1 (100)
Lung	5 (11.9)	1 (6.3)	2 (10.5)	2 (33.3)	0
Breast	2 (4.8)	0	1 (5.3)	0	1 (100)
Colorectal	1 (2.4)	0	1 (5.3)	0	0
Prostate	2 (4.8)	0	2 (10.5)	0	0
Pancreatic	3 (7.1)	2 (12.5)	1 (5.3)	0	0
Peritoneal	1 (2.4)	0	1 (5.3)	0	0
Cervical	2 (4.8)	2 (12.5)	0	0	0
Esophageal	2 (4.8)	1 (6.3)	1 (5.3)	0	0
Brain	2 (4.8)	1 (6.3)	0	1 (16.7)	0
Solid tumor stage					
Stage 3	7 (16.7)	1 (6.3)	5 (26.3)	1 (16.7)	0
Stage 4	12 (28.6)	6 (37.5)	4 (21.1)	1 (16.7)	1 (100)
Hematological malignancy	22 (52.4)	9 (56.3)	10 (52.6)	3 (50.0)	0
Lymphoma (aggressive)	5 (11.9)	2 (12.5)	3 (15.8)	0	0
Leukemia (acute)	4 (9.5)	1 (6.3)	2 (10.5)	1 (16.7)	0
Leukemia (chronic)	3 (7.1)	2 (12.5)	0	1 (16.7)	0
Myeloma	6 (14.3)	4 (25.0)	2 (10.5)	0	0
MDS	2 (4.8)	0	1 (5.3)	1 (16.7)	0
MDS/MPN overlap syndrome	1 (2.4)	0	1 (5.3)	0	0
Amyloid	1 (2.4)	0	1 (5.3)	0	0
Brain metastasis	2 (4.8)	1 (6.3)	1 (5.3)	0	0
Cancer treatment, *n* (%)					
Systemic treatment	40 (95.2)	15 (93.8)	18 (94.7)	6 (100)	1 (100)
Cytotoxic	24 (57.1)	7 (43.8)	13 (68.4)	3 (50.0)	1 (100)
Monoclonal antibody	3 (7.1)	1 (6.3)	2 (10.5)	0	0
Oral targeted agent	9 (21.4)	5 (31.3)	1 (5.3)	2 (33.3)	1 (100)
Combination cytotoxic and monoclonal antibody	6 (14.3)	2 (12.5)	3 (15.8)	1 (16.7)	0
Combination cytotoxic and immune checkpoint inhibitor	4 (9.5)	2 (12.5)	1 (5.3)	1 (16.7)	0
Surgical treatment	5 (11.9)	1 (6.3)	2 (10.5)	2 (33.3)	0
Surgery within 3 mo of CAT	1 (2.4)	0	0	1 (16.7)	0
Radiation therapy	12 (28.6)	5 (31.3)	6 (31.6)	1 (16.7)	0
Radiation within 3 mo of CAT	8 (19.0)	3 (18.8)	4 (21.1)	1 (16.7)	0
Cellular therapy within 3 mo of CAT (autologous stem cell transplant)	4 (9.5)	2 (12.5)	2 (10.5)	0	0
Comorbidities, *n* (%)					
Chronic kidney disease	8 (19.0)	2 (12.5)	3 (15.8)	3 (50.0)	0
Chronic liver disease	2 (4.8)	0	1 (5.3)	0	1 (100)
Previous VTE history	5 (11.9)	2 (12.5)	3 (15.8)	0	0
Previous major bleed	1 (2.4)	0	0	1 (16.7)	0
Bleeding disorder (ITP)	2 (4.8)	0	2 (10.5)	0	0
Type of VTE at diagnosis, *n* (%)					
Lower DVT	16 (38.1)^a^	7 (43.8)^b^	8 (42.1)	1 (16.7)^b^	0
Upper DVT	9 (21.4)	4 (25.0)	5 (26.3)^b^	0	0
Pulmonary embolism	17 (40.5)	5 (31.3)^b^	6 (31.6)^b^	6 (100)^b^	0
Other	3 (7.1)	1 (6.3)	1 (5.3)	0	1 (100)

CAT, cancer-associated thrombosis; DOAC, direct oral anticoagulant; DVT, deep vein thrombosis; ITP, immune thrombocytopenic purpura; LMWH, low molecular weight heparin; MDS, myelodysplastic syndrome; MPN, myeloproliferative neoplasm; UFH, unfractionated heparin; VTE, venous thromboembolism.

a There were 16 patients with lower extremity DVT at diagnosis, all of which were proximal. There were no patients with a distal lower extremity DVT.

b Some patients were diagnosed with multiple types of VTE concurrently, so the numbers indicated may not equal the total number of patients in the respective column.

Twenty patients (47.6%) had a solid organ malignancy, most commonly lung cancer (*n* = 5; 11.9%), while 22 (52.4%) had a hematologic malignancy, with multiple myeloma as the most frequent subtype (*n* = 6; 14.3%). Forty patients (95.2%) received systemic treatment, most often with cytotoxic therapy (*n* = 24; 57.1%). Other systemic cancer-directed agents included monoclonal antibodies (*n* = 3; 7.1%) and oral targeted agents (*n* = 9; 21.4%). Within 3 months of VTE diagnosis, 1 (2.4%) patient underwent surgery, 8 (19.0%) patients received radiation therapy, and 4 (9.5%) patients underwent autologous stem cell transplant.

### 3.2 Platelet count trends

The number of patients with thrombocytopenia during the time intervals assessed post-CAT diagnosis was as follows: days 1 to 7: 22 (52.4%); days 8 to 14: 19 (45.2%); days 15 to 30: 21 (50.0%); days 31 to 60: 20 (47.6%); and days 61 to 90: 19 (45.2%) (Table 2). In total, 11 (26.2%) patients had a platelet count <25,000/μL at some point within the 90-day period. Most of these severely thrombocytopenic patients had a hematologic malignancy (*n* = 9; 81.8%) including multiple myeloma (*n* = 2; 18.2%), aggressive lymphoma (*n* = 2; 18.2%), acute leukemia (n = 2; 18.2%), chronic leukemia (*n* = 1; 9.1%), and myelodysplastic syndrome (*n* = 2; 18.2%), while 2 patients had lung cancer (18.2%).

**Table 2:** Anticoagulant types and doses over 90 days post-CAT diagnosis in thrombocytopenic patients.

Time interval	Days 1-7			Days 8-14			Days 15-30			Days 31-60			Days 61-90		
Platelet Count (1000/μL)	<25	25-50	50-100	<25	50	50-100	<25	50	50-100	<25	50	50-100	<25	50	50-100
Number of occurrences^a^	3	9	19	4	6	16	4	10	17	5	10	16	4	9	15
Anticoagulant Dose															
DOAC	-	1	5	2	2	6	2	4	7	2	6	9	2	5	9
Full	-	-	5	1	2	6	1	3	6	1	4	7	-	2	8
Prophylactic	-	1	-	1	-	-	1	1	1	1	2	2	2	3	1
LMWH	2	5	9	-	3	6	1	3	5	2	3	7	1	2	4
Full	2	4	8	-	3	6	-	2	5	1	2	6	-	-	2
Intermediate	-	-	-	-	-	-	-	-	-	-	-	1	1	1	1
Prophylactic	-	1	1	-	-	-	1	1	-	1	1	-	-	1	1
UFH	1	3	5	-	-	-	-	-	-	-	-	-	-	-	-
Other^b^	-	-	-	1	-	1	-	-	-	-	-	-	-	-	-
None	-	-	-	1	1	3	1	3	5	1	1	-	1	2	2
**Total number of thrombocytopenic patients per time interval**	**22**			**19**			**21**			**20**			**19**		

CAT, cancer-associated thrombosis; DOAC, direct oral anticoagulant; LMWH, low molecular weight heparin; UFH, unfractionated heparin.

a The number of occurrences includes patients that had any of the applicable platelet counts (<25,000/μL, 25,000-50,000/μL, and/or 50,000-100,000/μL) for a given time interval. Some patients may have had fluctuating platelet counts that fell into more than one range within a certain time period. As such, the number of occurrences is not equivalent to the total number of thrombocytopenic patients per time interval.

b Two thrombocytopenic patients were receiving fondaparinux during days 8-14 and are represented by the “Other” category.

The number of thrombocytopenic patients with correlating platelet count range within the first 7 days of VTE diagnosis was: <25,000/μL, *n* = 3 (13.6%); 25,000 to 50,000/μL, *n* = 9 (40.1%); and 50,000 to 100,000/μL, *n* = 19 (86.3%). By the end of the first month post-CAT diagnosis (days 15-30), the number of thrombocytopenic patients with correlating platelet count range was: <25,000/μL, *n* = 4 (19.0%); 25,000 to 50,000/μL, *n* = 10 (47.6%); and 50,000 to 100,000/μL, *n* = 17 (81.0%) (Table 2).

Eighteen platelet transfusions took place among 9 patients throughout the 90-day follow-up period. Transfusion thresholds were known for 15 of the 18 transfusions given and ranged from 10,000 to 50,000/μL, most commonly 50,000/μL. Most transfusions occurred within the first month after VTE diagnosis (*n* = 12; 66.7%) compared to the second (*n* = 3; 16.7%) and third months (*n* = 3; 16.7%) (Table 3). There was no difference in transfusion frequency among patients receiving a DOAC (*n* = 7; 38.9%) or LMWH (n = 7; 38.9%). Of the 9 patients who received a platelet transfusion, the majority had a hematologic malignancy (*n* = 7; 77.8%) with diagnoses including aggressive lymphoma (*n* = 2; 22.2%), acute leukemia (*n* = 2; 22.2%), myelodysplastic syndrome (*n* = 2; 22.2%), and amyloidosis (*n* = 1; 11.1%). No platelet transfusion-related complications were documented within the 90-day follow-up period.

**Table 3:** Platelet transfusion data.

Time interval	Number of platelet transfusions	Average number of adult dosages received	Standard deviation (×1000 platelet/μL)	Average platelet transfusion threshold (×1000/μL)
Days 1-7	5	3.2	3.2	45
Days 8-14	4	3.3	2.6	50
Days 15-30	3	4.3	3.1	50
Days 31-60	3	2.7	1.5	10
Days 61-90	3	2.3	1.2	50
**Total**	**18**	**3.2**	**2.4**	**38**

### 3.3 Anticoagulant management

LMWH was the most common initial treatment choice (*n* = 19; 45.2%). The majority of patients received the full dose (*n* = 18; 42.9%), while 1 (2.4%) received a prophylactic dose. Sixteen (38.1%) patients initially received a DOAC, with the majority receiving the full dose (*n* = 14; 33.3%), while 2 (4.8%) received a reduced dose. Six (14.3%) patients were initially treated with unfractionated heparin (UFH). One patient did not receive up-front anticoagulant within the first 7 days of VTE diagnosis due to a gastrointestinal (GI) bleed but was eventually started on prophylactic dose LMWH after an interval restaging computed tomography scan demonstrated progression of the clot. Apixaban was the most common type of DOAC used initially (*n* = 12; 75.0%), while the most common type of LMWH was dalteparin (*n* = 18; 94.7%).

Several anticoagulant and dose changes took place across the 90-day follow-up period. Among the 19 individuals initially treated with LMWH, 13 patients later switched to a DOAC owing most often to patient preference for an oral anticoagulant or adherence issues with LMWH. No patients initially treated with a DOAC switched to LMWH. We recorded details of the first dose adjustment during each time interval for each patient. Of 48 of them, the most common reason for the first dose adjustment in each time interval was due to low platelets (14/48; 29.2%). The changes made due to low platelets were most often a dose reduction of the same anticoagulant (6/14; 42.9%) or discontinuation of anticoagulation altogether (6/14; 42.9%). Only 2 of the 6 patients whose anticoagulation was stopped due to low platelets were restarted within our follow-up period. Two of the 4 patients who were not restarted on anticoagulation during our follow-up period passed away due to progression of their malignancies shortly after discontinuation, and the other 2 did not resume due to ongoing thrombocytopenia <25,000/μL.

Over the 90-day follow-up, the number of thrombocytopenic patients treated with a DOAC increased from 5 to 12. These patients also varied in their degree of thrombocytopenia, with 2 severely thrombocytopenic patients (platelet count <25,000/μL) being treated with reduced-dose DOACs by the final month. Over this same interval, the number of thrombocytopenic patients treated with LMWH decreased from 9 to 5. The remaining 2 thrombocytopenic patients in days 61-90 did not receive anticoagulation.

### 3.4 Patient outcomes

In the entire cohort, 1 recurrent lower extremity DVT (*n* = 1/42; 2.4%) was noted in the second month in a patient treated with LMWH. The patient was not thrombocytopenic and was receiving dalteparin at 150 units/kg once daily as per the CLOT trial regimen [17]. Dalteparin dose reduction occurred 1 week prior to VTE recurrence. The patient did not have any other risk factors for recurrence including surgery, change in chemotherapy, or a change in their hormone therapy in the weeks leading up to the recurrence.

There were 8 bleeding events (8/42; 19.0%) within the 90-day follow-up period, none of which were major bleeds (Table 4). The most common bleeding type was clinically relevant nonmajor bleeding (CRNMB) (7/8; 87.5%), with the most common bleed location being gastrointestinal (4/8; 50.0%), and there was 1 (1/8; 12.5%) minor bleed. Among these bleeding events, 3 occurred in those receiving DOACs, 2 receiving LMWH, 1 receiving UFH, and 2 who were not receiving any anticoagulation.

**Table 4:** Bleeding events among patients with cancer-associated thrombosis and thrombocytopenia.

Time interval (d)	Bleed classification	Bleed location	Anticoagulant type	Dosage	Platelet count (×1000/μL)
1-7	Clinically relevant nonmajor bleed	GI	DOAC	Apixaban full	>100
1-7	Clinically relevant nonmajor bleed	MSK	UFH	NA	50-100
1-7	Clinically relevant nonmajor bleed	GI	None	NA	Unknown
8-14	Clinically relevant nonmajor bleed	GU	DOAC	Apixaban full	25-50
8-14	Clinically relevant nonmajor bleed	GI	None	NA	50-100
15-30	Clinically relevant nonmajor bleed	Mucocutaneous	LMWH	Dalteparin full	>100
31-60	Clinically relevant nonmajor bleed	Mucocutaneous	LMWH	Dalteparin full	25-50
31-60	Minor	GI	DOAC	Apixaban full	<25

DOAC, direct oral anticoagulant; GI, gastrointestinal; GU, genitourinary; LMWH, low molecular weight heparin; MSK, musculoskeletal; NA, not applicable; UFH, unfractionated heparin.

All patients who bled on a DOAC were receiving full-dose apixaban at the time. Two experienced CRNMB (1 GI and 1 genitourinary), and 1 minor GI bleed. The platelet count at the time of bleeding was variable, with 1 patient having a platelet count >100,000/μL, the second patient having a platelet count between 25,000 and 50,000/μL, and the third patient having a platelet count <25,000/μL. One of the patients, who had refractory lymphoma and was transitioned to end-of-life care, experienced rapid progressive decline and died 3 days after the bleed. As for those on LMWH, 2 patients had CRNMB (mucocutaneous). At the time of bleeding, 1 had a platelet count >100,000/μL, and the other had a platelet count 25,000 to 50,000/μL.

## 4 Discussion

In our cohort of patients with CAT and thrombocytopenia, anticoagulant dose and type changed frequently in the first 90 days. The overall rate of recurrent VTE in 90 days was 2.4%, and the rate of bleeding events was 19.0% (mostly CRNMB). Within the 90-day follow-up period in our study, there was 1 recurrent thrombotic event documented in a patient receiving LMWH, and no patient receiving DOAC experienced recurrent VTE. The type of VTE was similar between the different anticoagulant strategies used in our study, with the most common type of VTE being a pulmonary embolism. In a previous prospective, multicenter, observational cohort of patients with CAT and thrombocytopenia (Thrombocytopenia Related Outcomes with Venous thromboembolism [TROVE] study), patients treated with a DOAC did not develop recurrent VTE throughout the 60-day follow-up, although only 16 patients received a DOAC in this study [18]. Another prospective, multicenter cohort study, known as the CAVEaT study, also revealed that patients initially treated with a DOAC after CAT diagnosis did not develop recurrent VTE, although the sample size was also small, with only 4 patients receiving a DOAC up-front for CAT management [19].

Overall rates of recurrent VTE within 90 days after CAT diagnosis were lower in our study population compared to previously reported rates of >30% in other retrospective chart reviews [20]. However, the main comparator study by Kopolovic et al. [20], which similarly followed patients for 90 days and employed 100,000/μL as the platelet cutoff for inclusion, only studied hospitalized patients, and 23% of their patients did not receive anticoagulation owing to the site of index VTE (more commonly upper extremity DVT) or prolonged thrombocytopenia. Small patient numbers and differences in baseline patient characteristics also likely account for some of these variances.

Our study shows an overall similar number of bleeding events among patients treated with DOACs and LMWH. This contrasts with the results of a recent systematic review and meta-analysis, which identified 2 studies (TROVE and CAVEaT) that demonstrated higher rates of bleeding associated with DOACs compared to LMWH [21]. There were relatively fewer patients with luminal intestinal and genitourinary malignancies receiving DOACs in our study compared to those in the TROVE study, which may account for this difference, as the majority of bleeding events in the TROVE study were GI [18]. In the CAVEaT study, bleeding occurred in 50% of those initially managed with a DOAC, but this conclusion is severely limited by a small sample size, with only 2 patients experiencing a bleeding event. Moreover, these bleeding events occurred >4 weeks after initial diagnosis of CAT in patients with platelet counts of 40,000 to 50,000/μL [20], which most guidelines would identify as an acceptable platelet threshold for full-dose anticoagulant [8,9]. Most bleeding events in our study population occurred after the initial week of CAT diagnosis, at platelet count ranges >25,000/μL in the presence of full-dose anticoagulation, as well as 1 minor bleed in a patient receiving full-dose apixaban with platelets <25,000/μL. Guidelines suggest a modified dose anticoagulant strategy when platelet count is <50,000/μL [8,9]. In our cohort, most patients initially given a DOAC had a platelet count >50,000/μL, which could also explain the lower risk of bleeding.

The anticoagulation strategy was seemingly influenced by the severity of thrombocytopenia and time interval from CAT diagnosis. Only 1 of the 16 individuals who were initially prescribed a DOAC in the first 7 days after VTE had a platelet count <50,000/μL. However, over the course of the 90 days, the range of platelet counts for those receiving DOACs varied, with 5/11 (45.5%) patients with platelets <25,000/μL to 9/19 (47.4%) patients with platelets 25,000 to 50,000/μL treated with a DOAC. LMWH was the anticoagulant of choice for patients with CAT and thrombocytopenia within the first 14 days (about 2 weeks), after which more patients with CAT and thrombocytopenia were treated with DOACs than with LMWH, most commonly due to patient preference. This aligns with previous studies that identified a strong patient preference for the convenience of oral anticoagulants compared to daily injections [13,14]. Our study results are consistent with previous studies that have shown that patients with CAT and thrombocytopenia are often treated heterogeneously, in which severity of initial thrombosis and the duration of thrombocytopenia are relevant factors [20].

Almost all patients with a platelet count <25,000/μL had a hematologic malignancy, which is in keeping with previous studies demonstrating that patients with hematologic malignancy and CAT are at an increased risk of severe thrombocytopenia [6]. Risk of major bleeding and recurrent VTE has been shown to vary among patients with hematologic compared to solid organ malignancies [22], with hematologic malignancies generally associated with lower rates of VTE recurrence and increased bleeding depending on the diagnosis and treatment.

Our study is limited by the small sample size and inherent biases of a retrospective chart review, which lends itself to variability in baseline patient characteristics between the different anticoagulant treatment groups. Although the distribution of malignancy types and stage was similar among patients receiving DOACs and LMWH, more patients with certain hematologic malignancies associated with severe thrombocytopenia and increased bleed risk, such as acute leukemia, myelodysplastic syndrome, and aggressive lymphoma, received LMWH in our cohort review [6]. This may have underestimated the bleeding risk associated with DOACs.

Overall, our study suggests that DOACs may be a feasible anticoagulant strategy for selected patients with CAT and thrombocytopenia. While patients with platelets <100,000/μL were underrepresented in the major randomized controlled trials evaluating DOACs for CAT management, there were no adverse events identified in patients with platelets >50,000/μL receiving a DOAC in our study. Moreover, rates of bleeding were similar among patients receiving DOACs and LMWH with platelets between 25,000 to 50,000/μL later in their course of treatment, suggesting that consideration of a DOAC in the subacute treatment phase may be reasonable.

## Conclusion

Rates of recurrent venous thrombosis and bleeding were similar among thrombocytopenic patients with CAT treated with DOACs and LMWH in our cohort, although variation in baseline patient characteristics and the small number of patients remain major limitations. Further prospective research is needed to determine whether DOACs represent a suitable option for patients with concurrent CAT and thrombocytopenia.
